# Supplementary material for: Application of Bio-Active Elastin-like Polypeptide on Regulation of Human Mesenchymal Stem Cell Behavior
Source: Biomedicines. 2022 May 17;10(5):1151. doi: 10.3390/biomedicines10051151 (PMC9138580; doi:10.3390/biomedicines10051151)
Supplement: Supplementary file 1 [file biomedicines-10-01151-s001.zip › biomedicines-1582160-Supplementary.pdf]

Application of Bio-active Elastin-like Polypeptide on Regulation of Human Mesenchymal Stem Cell Behavior

Sarangthem Vijaya [1,2], Harshita Sharma [3], Mohini Mendiratta [4], Ranjit Kumar Sahoo [4], Rang-Woon Park [1,*], Lalit Kumar [4], Thoudam Debraj Singh [5,*] and Sujata Mohanty [3,*]

[1]Department of Biochemistry and Cell Biology, School of Medicine, Cell and Matrix Research Institute, Kyungpook National University, Daegu 41944, Korea; devi1703@gmail.com (V.S.); nwpark@knu.ac.kr (R-W. P)

[2]Department of Pathology, All India Institute of Medical Sciences, New Delhi 110029, India; devi1703@gmail.com (V.S.)

[3]Stem Cell Facility, DBT Centre of Excellence for Stem Cell Research, All India Institute of Medical Sciences, New Delhi 110029, India; sharshita558@gmail.com (H.S.)

[4]Department of Medical Oncology, All India Institute of Medical Sciences, New Delhi 110029, India; mohinimendiratta@gmail.com (M.M.); drranjitmd@gmail.com (R.K.S.); lalitaiims@yahoo.com (L.K.)

[5]Department of Medical Oncology Laboratory, All India Institute of Medical Sciences, New Delhi 110029, India; debraj.thoudam@gmail.com (T.D.S.)

***Correspondence:** nwpark@knu.ac.kr (R-W.P.); debraj.thoudam@gmail.com (T.D.S.); drmohantysujata@gmail.com (S.M.)

Methods

Characterization of thermal properties

Thermal responsive phase transition behaviour of fusion-ELPs were determined by monitoring the optical density of protein solutions at wavelength 350 nm as a function of temperature using Cary UV-visible spectrophotometer equipped with temperature controller (Agilent Technologies, Santa Clara, USA). The absorbance was monitored from 20 °C to 55 °C in 1°C /min increments. The transition temperature Tt defined as 50% turbidity of ELP solutions were estimated at a concentration of 25 μM.

Cell adhesion assay

A 96-well plate were coated with different concentrations (0.625, 1.25, 2.5 and 5 μM) of ELPs at 4 °C for overnight. Plates were washed with PBS for 2 times and uncoated surfaces were blocked with 1% BSA for 30 min at 37 °C. Human skin fibroblast cells (IHF) ($5x10^3$ cells) were seeded on protein coated plate and incubate for different time intervals (1, 3 and 6 h) at 37 °C. The unattached cells were removed by washing 3 times with PBS and attached cells were fixed with 4% paraformaldehyde for 15 min at room temperature. The plates were washed twice with PBS and cells were stained with Crystal Violet (Sigma Aldrich, St. Louis, USA) for 10 min. Excessive stains were removed through washing with PBS many times. The attached cells were observed under light microscope. The stain was extracted from each cell by resolubilizing with 10% acetic acid (Junsei Chemical Co. Ltd., Tokyo, Japan) and the absorbance was measured at 595 nm using ELISA reader.

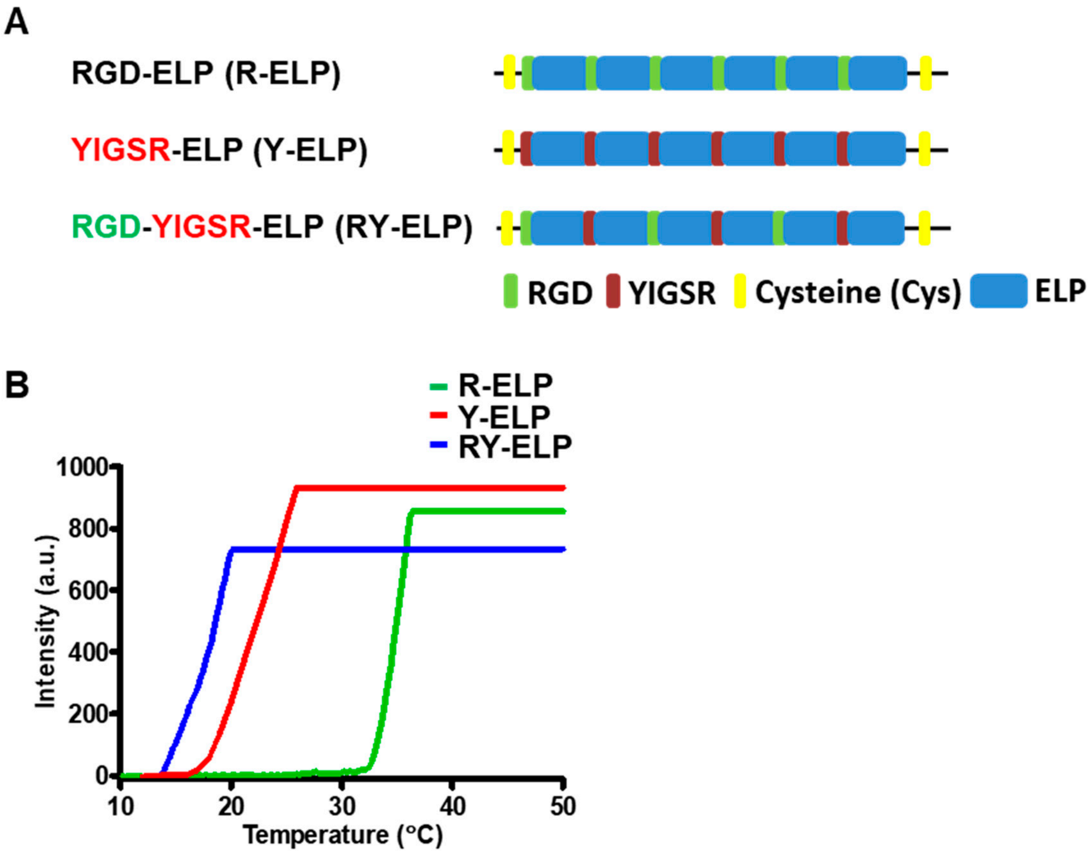

Figure S1. (A) Corresponding amino acid sequences of fusion ELPs. ECM binding domains (RGD and YIGSR) were incorporated periodically along with ELP sequences. (B) Temperature -dependent phase transition was observed for all the biopolymers. The Tt was found to be 34.71 ^{o}C, 21.5 ^{o}C and 22.21 ^{o}C for R-ELP, Y-ELP and RY-ELP respectively.

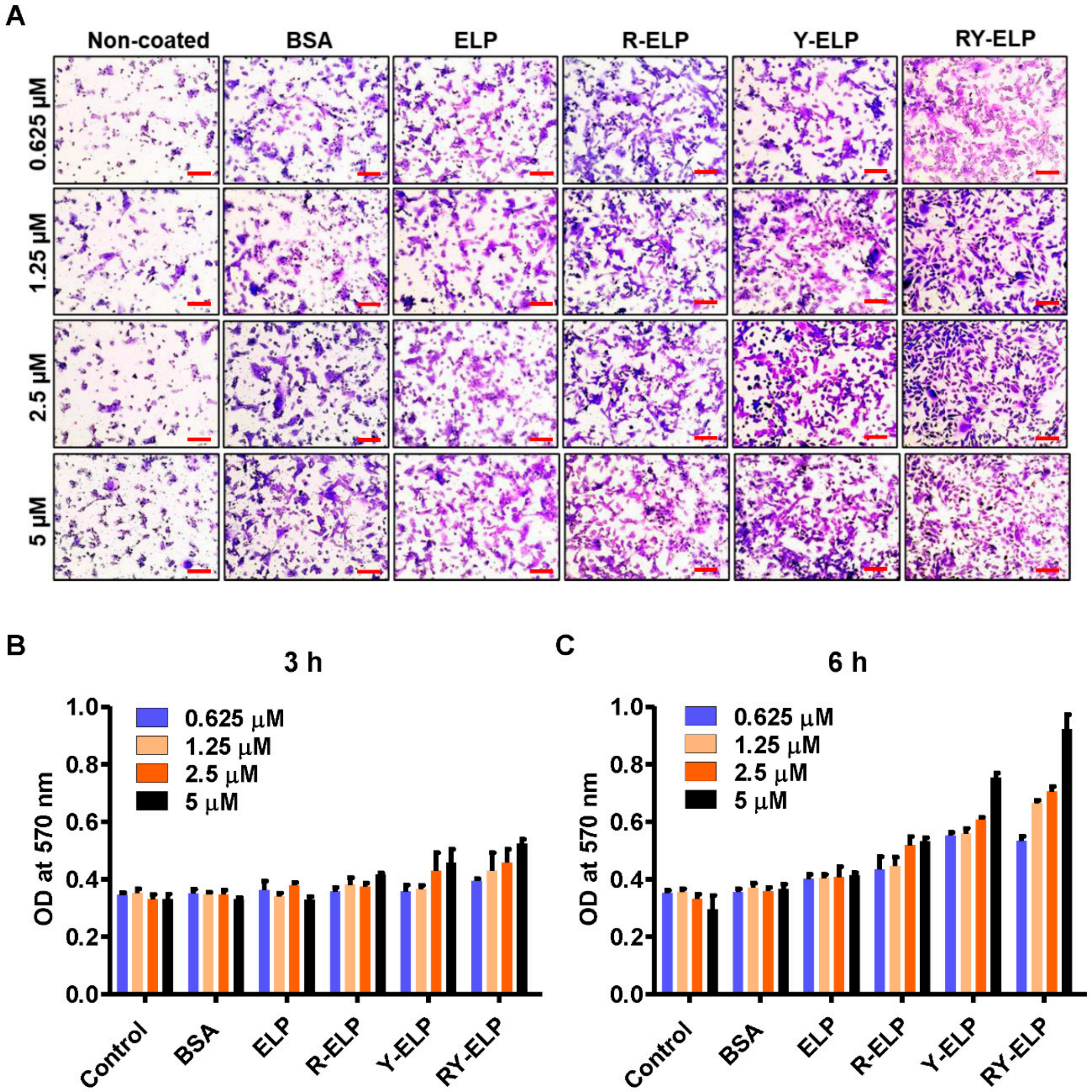

Figure S2. (A) Dose-dependent IHF cells adhesion was examined on fusion-ELP coated plates. Briefly, 96-well plate was coated with respective polymers overnight at 4°C. Then, 5× 10^3 IHF cells were seeded in each well and observed cell adherence for different time intervals. Attached cells were fixed and stained with crystal violet, then observed under bright field microscope (10 ×). The average number of attached cells per field was determined for various concentrations of fusion-ELPs (1.25, 2.5 and 5 μM) at 3 h (B) and 6 h (C). The stain was extracted from each cell by resolubilizing with 10% acetic acid and the absorbance was measured at 595 nm using plate reader. Results are presented as means±SDs (n = 3).

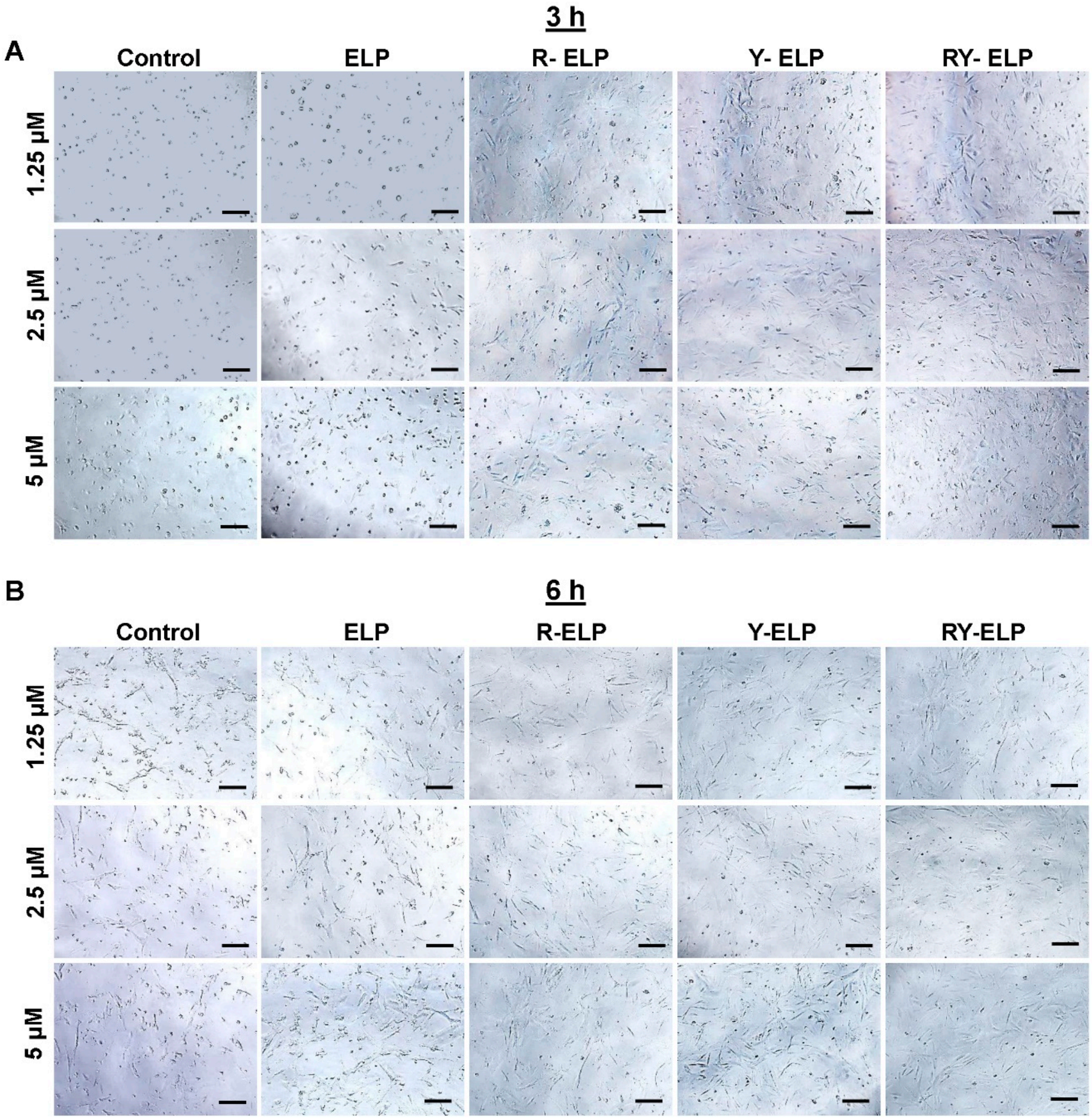

Figure S3. Representative images (×10) of adhered BM-MSCs cultured on fusion-ELPs modified surfaces. Time dependent cell attachment (A) 3 h and (B) 6 h, was monitored at different protein concentration (1.25, 2.5 and 5 μM) under phase-contrast light microscopy. The cells with "rounded" or less "spindle-like" morphology, indicating impaired attachment.

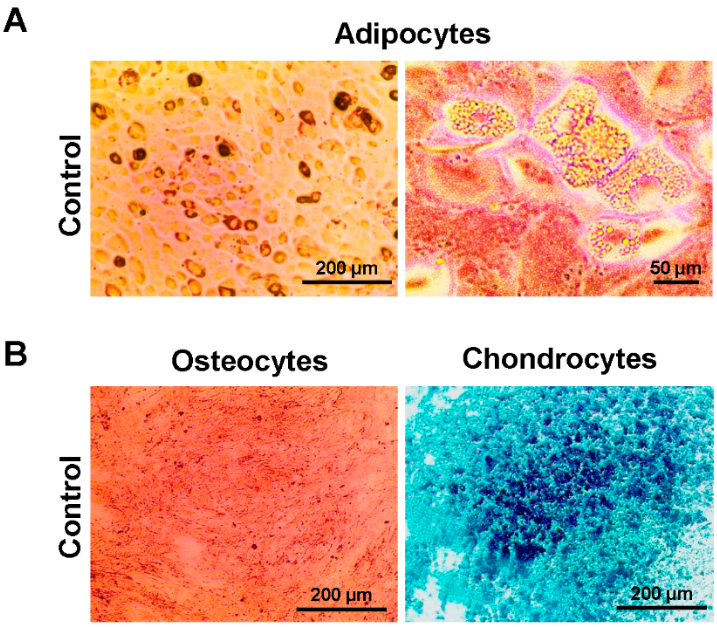

Figure S4. Trilineage staining of BMSC cultured on untreated surface. (A) Representative image of adipogenesis confirmed by cytochemical staining with Oil Red O. (B) Osteogenesis and chondrocyte differentiation of BM-MSCs were confirmed by Alzarin Red or Alcian Blue staining. Scale bar, 200 µm.

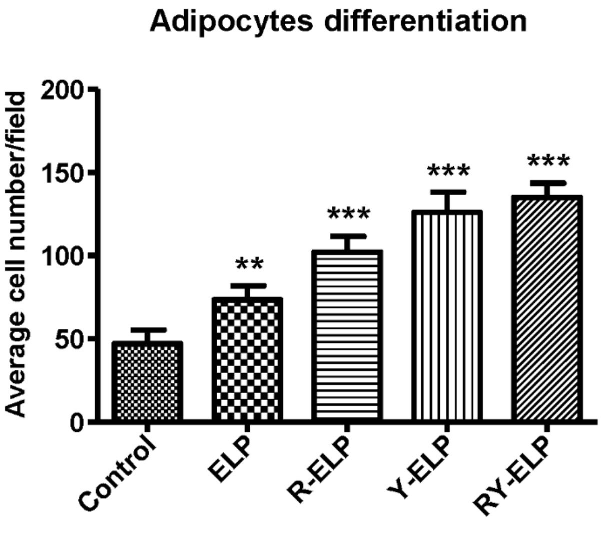

Figure S5. The graph represents the average number of adipocytes per field. ** P < 0.01 and *** P < 0.001, a statistically significant difference in cell adhesion activity of respective fusion-ELPs compared with uncoated control.

Table S1. The Mean and SD for the CD73, CD90 and CD105 expressions.

	CD 105		CD 90		CD 73	
	**Mean**	**SD**	**Mean**	**SD**	**Mean**	**SD**
**Untreated**	79.81	15.5	91.1	8.6	86.8	11.5
**R-ELP**	87.6	10.6	92.6	5.3	85.6	12.2
**Y-ELP**	87.04	9.2	89.46	8.5	87.2	9.4
**RY-ELP**	87.44	8.2	88.4	7.4	85.62	11.4
